# Alterations in platelet bioenergetics in Group 2 PH-HFpEF patients

**DOI:** 10.1371/journal.pone.0220490

**Published:** 2019-07-31

**Authors:** Quyen L. Nguyen, Yinna Wang, Nicole Helbling, Marc A. Simon, Sruti Shiva

**Affiliations:** 1 Division of Pulmonary Allergy and Critical Care Medicine, University of Pittsburgh, Pittsburgh, Pennsylvania, United States of America; 2 Vascular Medicine Institute, University of Pittsburgh, Pittsburgh, Pennsylvania, United States of America; 3 Division of Cardiology, University of Pittsburgh, Pittsburgh, Pennsylvania, United States of America; 4 Department of Pharmacology & Chemical Biology, Center for Metabolism & Mitochondrial Medicine, University of Pittsburgh, Pittsburgh, Pennsylvania, United States of America; University of Alabama at Birmingham, UNITED STATES

## Abstract

**Background:**

Pulmonary hypertension (PH) is characterized by elevated pulmonary artery pressure but classified into subgroups based on disease etiology. It is established that systemic bioenergetic dysfunction contributes to the pathogenesis of pulmonary arterial hypertension classified as World Health Organization (WHO) Group 1. Consistent with this, we previously showed that platelets from Group 1 PH patients demonstrate increased glycolysis and enhanced maximal capacity for oxidative phosphorylation, which is due to increased fatty acid oxidation (FAO). However, it remains unclear whether identical mitochondrial alterations contribute to the pathology of other PH subgroups. The most prevalent subgroup of PH is WHO Group 2, which encompasses pulmonary venous hypertension secondary to left heart disease. Here, we hypothesized that platelets from Group 2 subjects show bioenergetic alteration compared to controls, and that these changes were similar to Group 1 PH patients.

**Method and results:**

We isolated platelets from subjects with Group 2 PH and controls (n = 20) and measured platelet bioenergetics as well as hemodynamic parameters. We demonstrate that Group 2 PH platelets do not show a change in glycolytic rate but do demonstrate enhanced maximal capacity of respiration due at least partially to increased FAO. Moreover, this enhanced maximal capacity correlates negatively with right ventricular stroke work index and is not changed by administration of inhaled nitrite, a modulator of pulmonary hemodynamics.

**Conclusions:**

These data demonstrate that Group 2 PH subjects have altered bioenergetic function though this alteration is not identical to that of Group 1 PH. The implications of this alteration for disease pathogenesis will be discussed.

## Introduction

Pulmonary hypertension (PH), a condition characterized by elevated pulmonary artery pressures and vascular resistance, is classified into subgroups based on distinct etiologies, diagnostic criteria, and treatment strategies [1]. World Health Organization (WHO) Group 1 disease comprises pre-capillary pulmonary arterial hypertension (PAH), which may be idiopathic, inherited, or associated with collagen vascular disease, toxin exposure, or HIV infection. The most prevalent form of PH is Group 2 which encompasses post-capillary (either isolated post-capillary or combined post- and pre-capillary) pulmonary hypertension secondary to left heart disease, including valvular disease and heart failure. Group 3 PH develops in the setting of hypoxic lung disease. Group 4 represents chronic thromboembolic pulmonary hypertension. Finally, Group 5 PH develops by pathophysiology not captured by the previous subgroups such as post-splenectomy or in the setting of sarcoidosis where PH may develop by multiple mechanisms [1].

Due to the diverse etiologies of PH, a unifying causal molecular mechanism has been elusive. Altered cell metabolism in multiple tissues, propagated by mitochondrial dysfunction, is well-recognized to contribute to disease pathogenesis in animal models and in human patients with prototypical PAH who are classified within WHO Group 1 [2, 3]. Within pulmonary vascular cells, a switch to aerobic glycolysis confers apoptosis resistance and cellular hyperproliferation which contributes to vascular remodeling in PAH [4–8]. Outside of the pulmonary vasculature, cardiac and skeletal muscle display derangements in substrate metabolism which compounds the heart failure and exercise intolerance in PAH [9–14]. Using platelets as a less-invasive source of human mitochondria for assessment of bioenergetics, our group previously showed that platelets from Group 1 PAH patients mirror the enhanced glycolysis seen in other tissues, but also exhibit increased mitochondrial reserve respiratory capacity, which was associated with a switch to fatty acid oxidation (FAO) and correlated with hemodynamic changes [15]. Thus, dysregulation of mitochondrial metabolism represents a key feature in the pathobiology of Group 1 PAH. However, it has not been firmly established whether the mitochondrial dysfunction seen in Group 1 PAH is common to other PH subgroups and therefore could represent a common mechanistic link in all types of PH.

Far more prevalent than Group 1 disease, Group 2 PH is an increasingly recognized cause of morbidity and mortality, particularly in patients with the metabolic syndrome, which is characterized by obesity, insulin resistance, systemic hypertension, and dyslipidemia [16–18]. Some of these patients go on to develop heart failure with preserved ejection fraction and associated PH (HFpEF-PH) [19, 20]. Currently there is no FDA-approved treatment for Group 2 PH [21], highlighting an unmet need for a growing population. Recently, our group showed hemodynamic improvements in patients with Groups 1 and 2 PH following treatment with inhaled nitrite, a potent vasodilator, which has also been shown to regulate mitochondrial function [22]. Notably, nitrite treatment mediated greater hemodynamic responses in Group 2 patients compared to Group 1 PAH patients. Specifically, Group 2 patients showed a greater decrease in right atrial pressure (RAP), pulmonary capillary wedge pressure (PCWP), right ventricular (RV), and pulmonary artery (PA) pressures as well as increased pulmonary capillary compliance. Given our previous finding that altered platelet bioenergetics correlate with Group 1 PAH hemodynamics, we sought to determine 1) whether platelets from Group 2 PH patients would also show alterations in platelet bioenergetics compared to healthy individuals, 2) whether these differences would be identical to those observed in Group 1 PH, and 2) whether the differences in hemodynamic responses after nitrite treatment would translate to differential platelet mitochondrial responses.

## Materials and methods

### Study population

The PH groups consisted of patients classified as WHO Group 2, based on [1], who participated in the clinical trial “A Dose Escalation Study to Evaluate the Effect of Inhaled Nitrite on Cardiopulmonary Hemodynamics in Subjects With Pulmonary Hypertension” (ClinicalTrials.gov Identifier: NCT01431313) conducted at the University of Pittsburgh Medical Center [22]. The control group consisted of healthy age and gender matched subjects without known cardiopulmonary or hematologic disease. In the PH groups, hemodynamic parameters were derived from right heart catheterization performed on the same day as platelet bioenergetic assessment. The protocol for administration of inhaled nitrite to PH subjects is described in [22].

### Platelet isolation

Platelets were isolated by differential centrifugation and number quantified as previously described [23, 24]. Briefly, venous blood was collected in citrate by standard venipuncture. Whole blood was centrifuged (150*g*; 10 min) in the presence of PGI2 (1 μg/mL) to obtain platelet-rich plasma (PRP). Platelets were pelleted from PRP by centrifugation (1500*g*; 10 min), washed in erythrocyte lysis buffer containing PGI2, then resuspended in modified Tyrode’s buffer (20 mmol/L HEPES, 128 mmol/L NaCl, 12 mmol/L bicarbonate, 0.4 mmol/L NaH_2_PO_2_, 5 mmol/L glucose, 1 mmol/L MgCl_2_, 2.8 mmol/L KCl, pH 7.4). CD41a expression measured by flow cytometry was used to confirm that platelets were >99% pure.

### Measurement of platelet bioenergetics

Oxygen consumption rate (OCR) and extracellular acidification rate (ECAR) were measured in isolated platelets (50 x 10^6^/well) by extracellular flux analysis (XF24, Seahorse Biosciences, Billerica, MA) as previously described [23]. After measurement of basal OCR, OCR due to proton leak was determined by oligomycin A (2.5 μmol/L) treatment. Maximal uncoupled OCR was measured by the addition of the uncoupler carbonyl cyanide *p*-(trifluoro-methoxy) phenyl-hydrazone (FCCP; 0.7 μmol/L). Non-mitochondrial OCR (defined as the oxygen consumption rate of all cellular processes excluding mitochondrial respiration) was measured in the presence of rotenone (10 μmol/L). In a subset of samples, etomoxir (200 μmol/L) was added to quantify FAO-dependent OCR and 2-deoxyglucose (2-DG, 100 mmol/L) was added to quantify the glucose-oxidation-dependent OCR. Glycolytic rate was calculated by determining extracellular acidification rate (ECAR).

### Mitochondrial ROS generation

Platelets were incubated with MitoSOX (Invitrogen, Carlsbad, CA; 5 μM, 10 minutes). Fluorescent intensity was measured kinetically at 510/580 nm.

### Platelet activation

Platelet activation was performed as previously described [23]. Platelets were incubated with phycoerythrin (PE)-labeled mouse anti-human CD41a antibody and allophycocyanin (APC)-labeled mouse and antihuman CD62 antibody (30 min, 25°C) to measure surface *p*-selectin expression by flow cytometry (LSRFortessa with FASCDiva software; Becton Dickinson). Platelets were identified by their characteristic light scatter and CD41a antibody binding. Activated platelets are represented as percentage of 10,000 CD41a+ platelets exhibiting APC-CD62P fluorescence.

### Mitochondrial enzyme expression

Mitochondrial protein expression was measured by Western blot as previously described [23]. Integrin αIIβ antibody (sc-166599) was purchased from Santa Cruz Biotech, Dallas, TX. Antibodies for pyruvate dehydrogenase kinase 1 (PDK1 ab110335) and carnitine palmitoyltransferase-1 (CPT1 ab128568) were purchased from Abcam, Cambridge, MA.

### ETC complex and carnitine palmitoyltransferase-1 (CPT1) activities

Following several cycles of freeze/thaw, CPT1 activity of isolated platelets was determined by spectrophotometrically monitoring the generation of CoA-SH from 100 μM palmitoyl-CoA in the presence of 5 mM L-carnitine and 200 μM 5,5’-dinitro-bis-(2-nitrobenzoic acid) (DTNB) at an absorbance of 412 nm, as adapted from [25]. Enzymatic activity of complexes I, II, IV, and citrate synthase was spectrophotometrically measured as previously described [26].

### Statistics

Statistics were performed on Graphpad Prism 8.0 (La Jolla, CA) and IBM SPSS 24 (Armonk, NY). Data were compared by Student *t*-test or analysis of variance (ANOVA) where appropriate. Correlations were determined by 2-tailed Pearson’s correlation and linear regression analysis with 95% confidence interval. *P* < 0.05 was considered significant.

### Study approval

This study was approved by the Institutional Review Board of the University of Pittsburgh Medical Center (UPMC), and written informed consent was obtained from all subjects.

## Results

### Platelets from Group 2 PH patients exhibit altered mitochondrial bioenergetics and enzymatic activity

**Fig 1:**
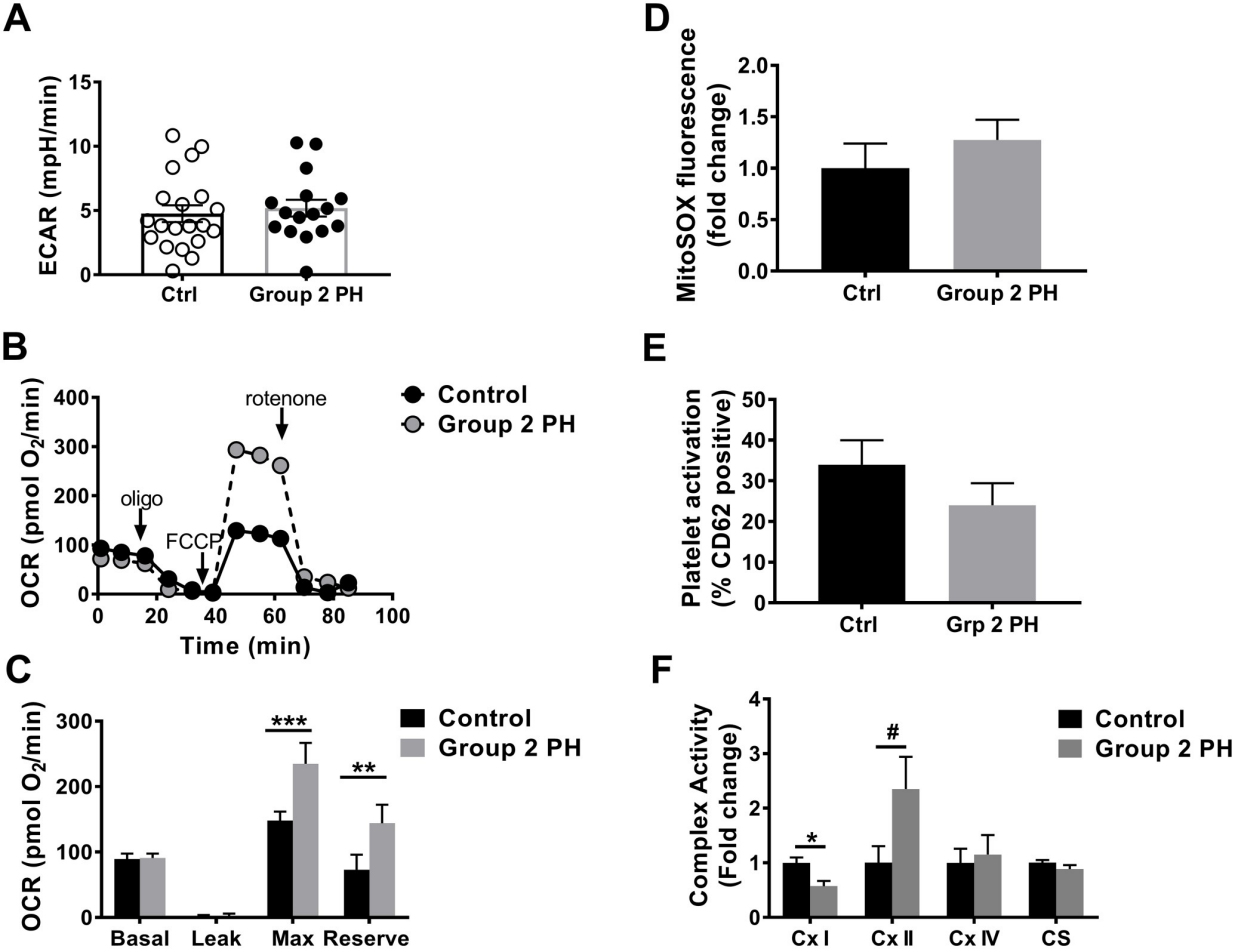
Platelets from Group 2 PH patients exhibit altered mitochondrial bioenergetics. **(A)** Basal glycolytic rate, measured by ECAR in control (n = 20) and Group 2 PH (n = 20) subjects. **(B)** Representative platelet OCR profiles from a healthy control subject and a patient with Group 2 PH. **(C)** Quantification of individual components of the platelet OCR profile in control (n = 20) and Group 2 PH (n = 20) subjects. **(D)** Mitochondrial superoxide production, measured by MitoSOX fluorescence, in control (n = 12) and Group 2 PH (n = 17) platelets. **(E)** % activated platelets, as measured by CD62 positivity, in control (n = 11) and Group 2 PH (n = 10) subjects. **(F)** Enzymatic activity (fold change of control) of mitochondrial complexes (Cx) I, II, IV, and citrate synthase (CS) from control (n = 5–7) and Group 2 PH (n = 5–11) subjects. Data are mean ± SEM. Unpaired 2-tailed t-test was used to compare groups.***p < 0.001, **p < 0.01, #p < 0.1.

We compared bioenergetics in platelets isolated from Group 2 HFpEF-PH patients (n = 20) with those from age- and gender- matched healthy control subjects (n = 20, Table 1). Measurement of platelet extracellular acidification rate (ECAR) showed that platelets from Group 2 PH subjects had no change in basal glycolytic rate compared to healthy controls (5.19 ± 0.66 vs. 4.75 ± 0.65 mpH/min, *p* = 0.64, Fig 1A). We next determined whether Group 2 PH patients showed changes in mitochondrial function by measuring the platelet oxygen consumption rate (OCR; Fig 1B and 1C). After correction for the non-mitochondrial OCR (subtraction of the rotenone-insensitive OCR), Group 2 PH platelets showed no significant difference compared to healthy controls in basal OCR (91.0 ± 6.66 vs. 89.2 ± 8.3 pmol/min, *p* = 0.9, Fig 1C) or proton leak (2.41 ± 3.56 vs. 0.56 ± 3.10 pmol O_2_/min, *p* = 0.9, Fig 1C). However, maximal OCR as well as reserve respiratory capacity (maximal—basal OCR) were significantly increased compared to controls (234.9 ± 31.9 vs. 147.8 ± 14.1 pmol O_2_/min, *p* <0.001 for maximal OCR; 143.9 ± 28.6 vs. 72.9 ± 23.3 pmol O_2_/min, *p* <0.01 for reserve OCR, Fig 1C). Notably, despite these differences in bioenergetics, no significant difference was observed between Group 2 PH platelets and healthy controls with respect to mitochondrial oxidant production (1.27 ± 0.2-fold vs. controls, *p* = 0.38, Fig 1D) or platelet activation as measured by surface p-selectin expression (15.7 ± 4.0% vs 25.8 ± 4.9% activated in controls, *p* = 0.12, Fig 1E). To determine whether mitochondrial enzymatic activity was increased in Group 2 PH platelets, we next examined the activities of electron transport chain (ETC) complexes (Fig 1F). Complex I activity was significantly lower in Group 2 PH platelets compared to control (0.57 ± 0.01-fold vs control, *p* = 0.013, Fig 1F). While it did not reach statistical significance, there was a trend to increased complex II activity, which was 2.35 ± 0.59-fold increased in Group 2 PH platelets compared to controls (*p* = 0.09, Fig 1F). Citrate synthase activity was unchanged in Group 2 PH platelets compared to controls, reflecting no significant differences in mitochondrial numbers (Fig 1F).

**Table 1:** Clinical characteristics of subjects.

	Control (n = 20)	Group 2 PH (n = 20)
**Gender** (% female)	50	50
**Age** (years)	69.4 (17.6)	69 (7.4)
**BMI** (kg/m^2^)		39.4 ± 10.7
**Comorbidities (%)**		
Diabetes		13 (65)
Systemic Hypertension		17 (85)
**Medication Use (%)**		
PDE5i		2 (10)
ERA		0 (0)
Prostacyclin		0 (0)
Aspirin		11 (55)
Metformin		5 (25)
Sulfonylurea		4 (20)
Insulin		10 (50)
**Baseline Hemodynamics**		
RAP (mm Hg)		12.2 ± 4.7
mPAP (mm Hg)		38.5 ± 8.0
PCWP (mm Hg)		20.1 ± 4.6
CO (L/min)		5.6 ± 1.9
CI (L/min/m^2^)		2.6 ± 0.9
PVR (WU)		3.8 ± 2.7
RV SWI (g-m/m^2^/beat)		14.56 ± 5.1

Data are mean ± SD. PDE5i: phosphodiesterase 5 inhibitor; ERA: endothelin receptor agonist; RAP: right atrial pressure; mPAP: mean pulmonary artery pressure; PCWP: pulmonary capillary wedge pressure; CO: cardiac output by thermodilution; CI: cardiac index; PVR: pulmonary vascular resistance; WU: Wood units; RV SWI: right ventricular stroke work index.

**Fig 2:**
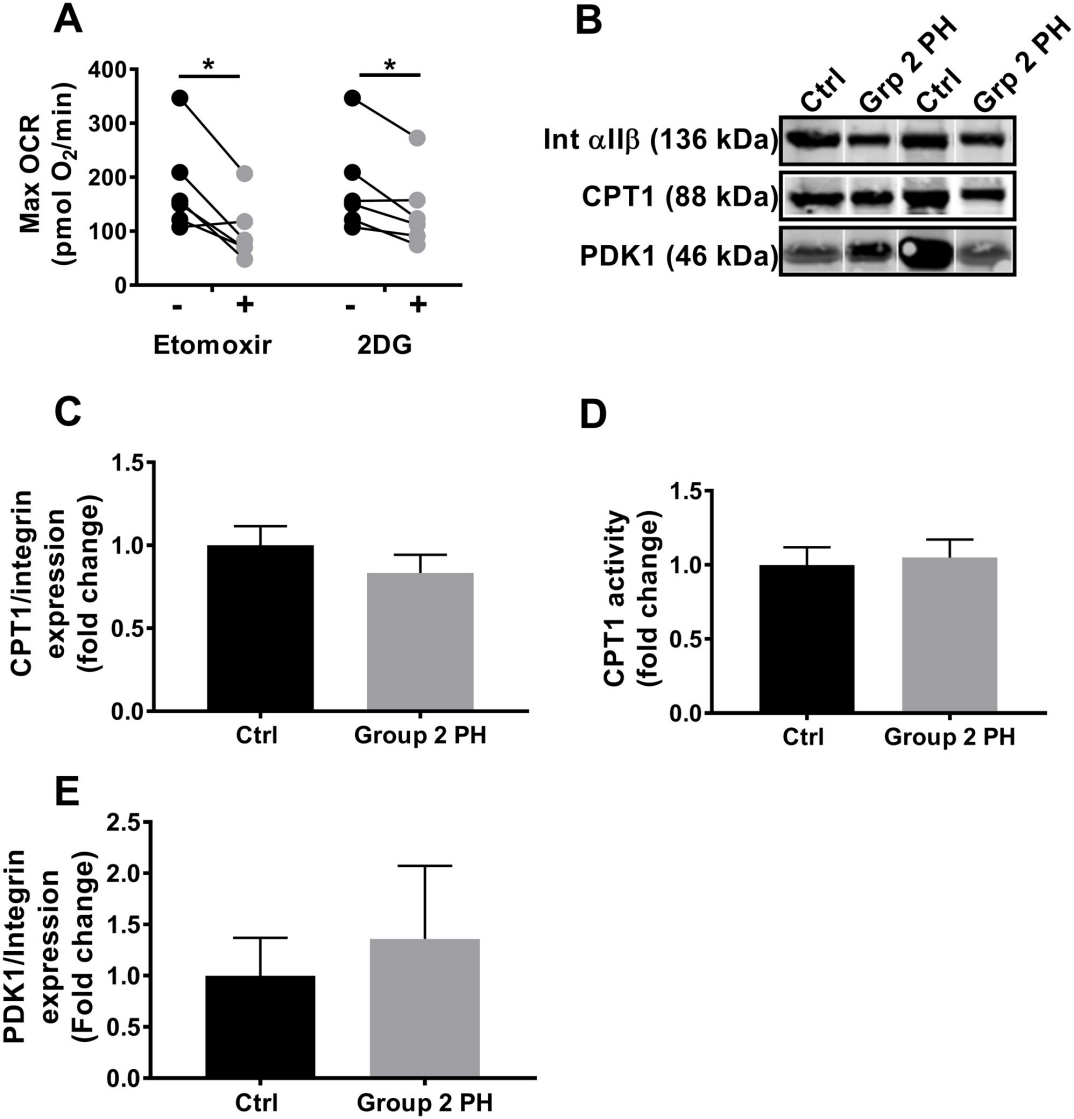
Fatty acid and glucose oxidation contribute to enhanced OCR in Group 2 PH Platelets. **(A)** Maximal OCR in platelets from Group 2 PH subjects (n = 6) untreated and treated with etomoxir or 2-deoxyglucose (2DG). Paired 2-tailed *t*-test was used to compare groups. **(B)** Representative western blots for integrin αIIβ, CPT1, and PDK1 in platelets from control and Group 2 PH subjects. **(C)** Quantification of CPT1/integrin αIIβ protein expression in platelets from control (n = 4) and Group 2 PH (n = 4) patients. Data are mean ± SEM. Unpaired 2-tailed *t*-test used to compare groups. **(D)** Enzymatic activity of platelet CPT1 in control (n = 9) and Group 2 PH (n = 11) subjects. Data are mean ± SEM. Unpaired 2-tailed *t*-test used to compare groups. **(E)** Quantification of PDK1/integrin αIIβ protein expression in platelets from control (n = 4) and Group 2 PH (n = 4) patients. Data are mean ± SEM. Unpaired 2-tailed *t*-test was used to compare groups. *p < 0.05.

Since we previously demonstrated that fatty acid oxidation (FAO) underlies the increase in maximal OCR in Group 1 PH platelets and complex II increases are often indicative of a substrate switch, we next sought to determine the contribution of FAO to maximal OCR in the Group 2 subjects. A subset of Group 2 PH platelets was treated with etomoxir (200 μmol/L), which inhibits carnitine palmitoyl transferase 1 (CPT1), a key regulating enzyme for FAO, prior to measurement of maximal OCR. Treatment of Group 2 PH platelets with etomoxir significantly diminished the maximal OCR (99.4 ± 23.4 vs. 192.1 ± 35.6 pmol O_2_/min without etomoxir, *p* = 0.013, Fig 2A), suggesting that FAO contributed to the enhanced respiratory capacity, similar to what we previously observed in Group 1 PAH platelets. Additionally, the same platelets were treated with 2-deoxyglucose (2DG; 100 mmol/L) to inhibit glycolysis prior to measuring the contribution of glucose oxidation to maximal OCR. The small but significant decrease in maximal OCR in the presence of 2DG demonstrated that glucose oxidation also contributed to Group 2 PH platelet maximal OCR, albeit to a lesser degree than FAO (138.7 ± 29.2 vs. 192.1 ± 35.6 pmol/min without 2DG, *p* = 0.022, Fig 2A). To determine the mechanism of increased FAO, we next examined CPT1 expression and activity. However, unlike Group 1 PH platelets, Group 2 PH platelets showed no significant change in protein expression (Fig 2B and 2C) nor the enzymatic activity of CPT1 (1.18 ± 0.17-fold vs. controls, *p* = 0.78, Fig 2D). Protein expression of pyruvate dehydrogenase kinase 1 (PDK1), which regulates entry of glucose toward oxidative phosphorylation, was also unchanged in Group 2 PH platelets (Fig 2B and 2E).

### Group 2 PH platelet bioenergetics correlate with RV dysfunction

**Fig 3:**
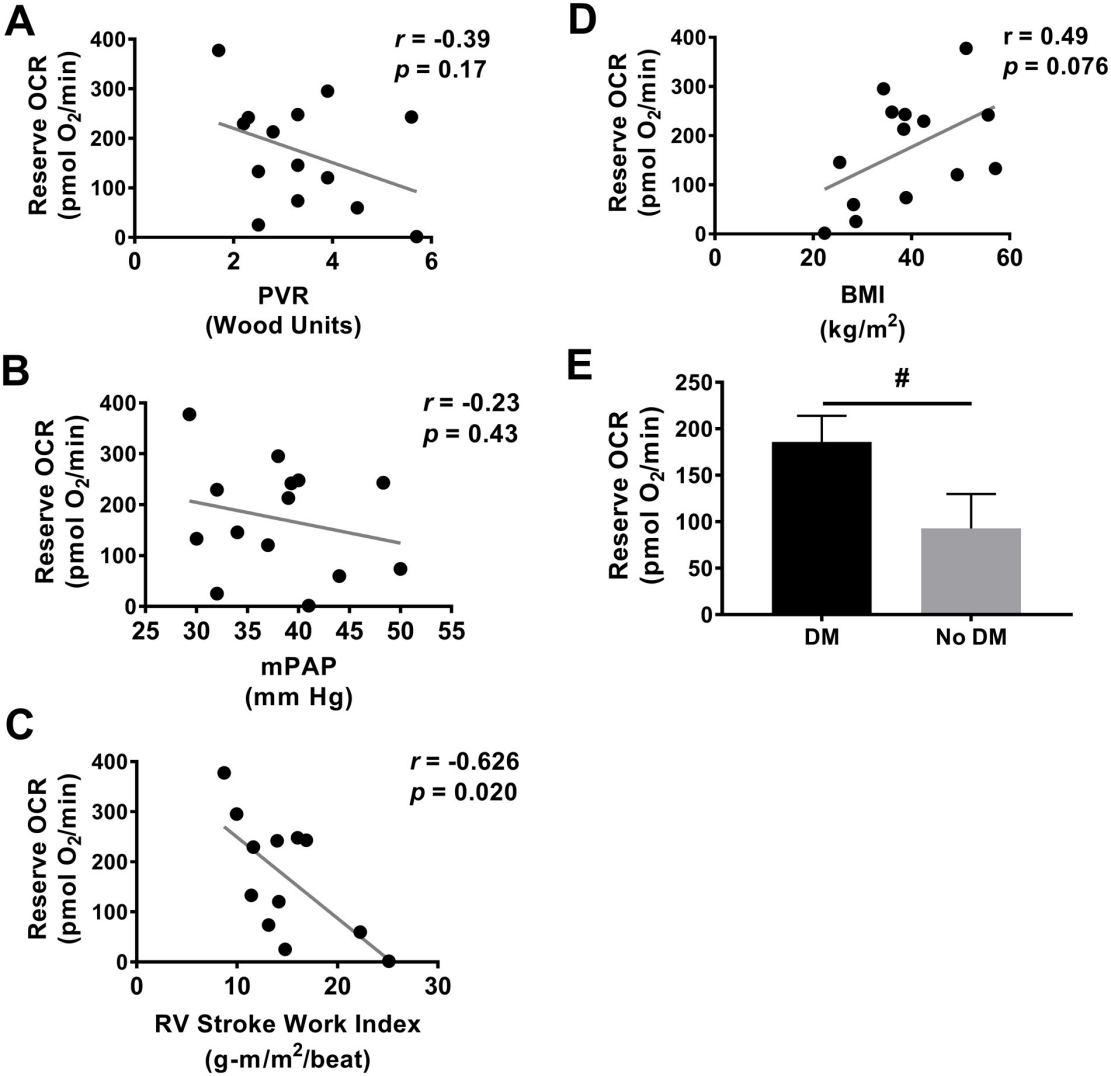
Group 2 PH platelet bioenergetics correlates with RV dysfunction. Pearson’s correlation of platelet reserve oxygen consumption rate with **(A)** pulmonary vascular resistance (PVR), **(B)** mean pulmonary artery pressure (mPAP), **(C)** right ventricular stroke work index (RV SWI), **(D)** body mass index (BMI). **(E)** Platelet reserve oxygen consumption rate in subject with and without diabetes mellitus (DM). Data are mean ± SEM. Unpaired 2-tailed *t*-test used to compare groups, #*p* < 0.1.

We previously showed a significant positive correlation in Group 1 PAH patients between platelet reserve OCR and more severe hemodynamic derangements, including mean pulmonary artery pressure (mPAP), pulmonary vascular resistance (PVR), and RV stroke work index (RV SWI). Compared to our Group 1 PAH cohort, Group 2 PH is hemodynamically characterized by more modest elevations in PVR (Table 1, [15]). In accordance, Pearson’s correlation did not yield significant associations between Group 2 PH platelet reserve OCR and patient PVR (r = -0.39, *p* = 0.17, Fig 3A) nor mPAP (r = -0.23, *p* = 0.42, Fig 3B). Interestingly, there was a significant negative correlation between RV SWI and platelet reserve respiration (r = -0.66, *p* = 0.02, Fig 3C) in Group 2 PH patients.

Many patients with PH-HFpEF show characteristics of metabolic syndrome. Thus, we next determined whether altered platelet bioenergetics were associated with obesity and diabetes. While it did not reach statistical significance, there was a trend toward BMI correlation with reserve OCR (Pearson r = 0.49, *p* = 0.076, Fig 3D). When reserve OCR was stratified by the presence of diabetes, reserve OCR was higher in diabetic subjects compared to non-diabetic Group 2 PH subjects, though this was not statistically significant, (185.9 ± 28.1 vs 92.81 ± 36.69 pmol/min, *p* = 0.069, Fig 3E). Notably, a number of Group 2 PH subjects were treated with diabetic medications (Table 1), which may affect cellular metabolism. By univariate regression, we did not find reserve OCR to be significantly influenced by the use of insulin (β = 0.38, *p* = 0.15), metformin (β = 0.110, *p* = 0.68), sulfonylureas (β = -0.09, *p* = 0.73), nor sitagliptin (β = -0.12, *p* = 0.67).

HFpEF-PH patients span a hemodynamic continuum encompassing those with isolated post-capillary PH (PVR ≤ 3 Wood units) and those who exhibit pre-capillary features (combined post- and pre-capillary PH, PVR > 3 Wood units) [21]. To determine whether platelet metabolism profiles can distinguish between patients with post-capillary PH and combined post- and pre-capillary PH, we stratified platelet mitochondrial parameters by PVR. Compared to those with PVR ≥ 3 Wood units (n = 10), platelets from patients with PVR < 3 Wood units (n = 10) did not significantly differ by basal OCR, maximal OCR, ECAR, or ROS production (Table 2).

**Table 2:** Degree of precapillary PH does not affect WHO Group 2 PH platelet bioenergetics.

	Pc-PH (PVR ≤ 3 WU)	CPC-PH (PVR > 3 WU)	*p*-value
**Basal OCR** (pmol/min)	92.1 ± 5.58	91.1 ± 11.58	0.94
**Maximal OCR** (pmol/min)	264.3 ± 37.7	228.7 ± 41.4	0.53
**ECAR** (mpH/min)	5.84 ± 0.80	4.99 ± 0.99	0.51
**Reserve OCR (pmol/min)**	172.2 ± 35.3	137.6 ± 34.3	0.49
**MitoSOX** (FL/min/mg)	0.044 ± 0.008	0.069 ± 0.02	0.18

Comparison of platelet bioenergetics in patients with isolated post-capillary PH (Pc-PH, defined by PVR ≤ 3 WU, n = 10) and combined pre- and post-capillary PH (Cpc-PH, defined by PVR > 3 WU), n = 10. Data are mean ± SEM. Unpaired 2-tailed *t*-test was used to compare groups.

### Nitrite does not affect Group 2 PH platelet respiration

**Fig 4:**
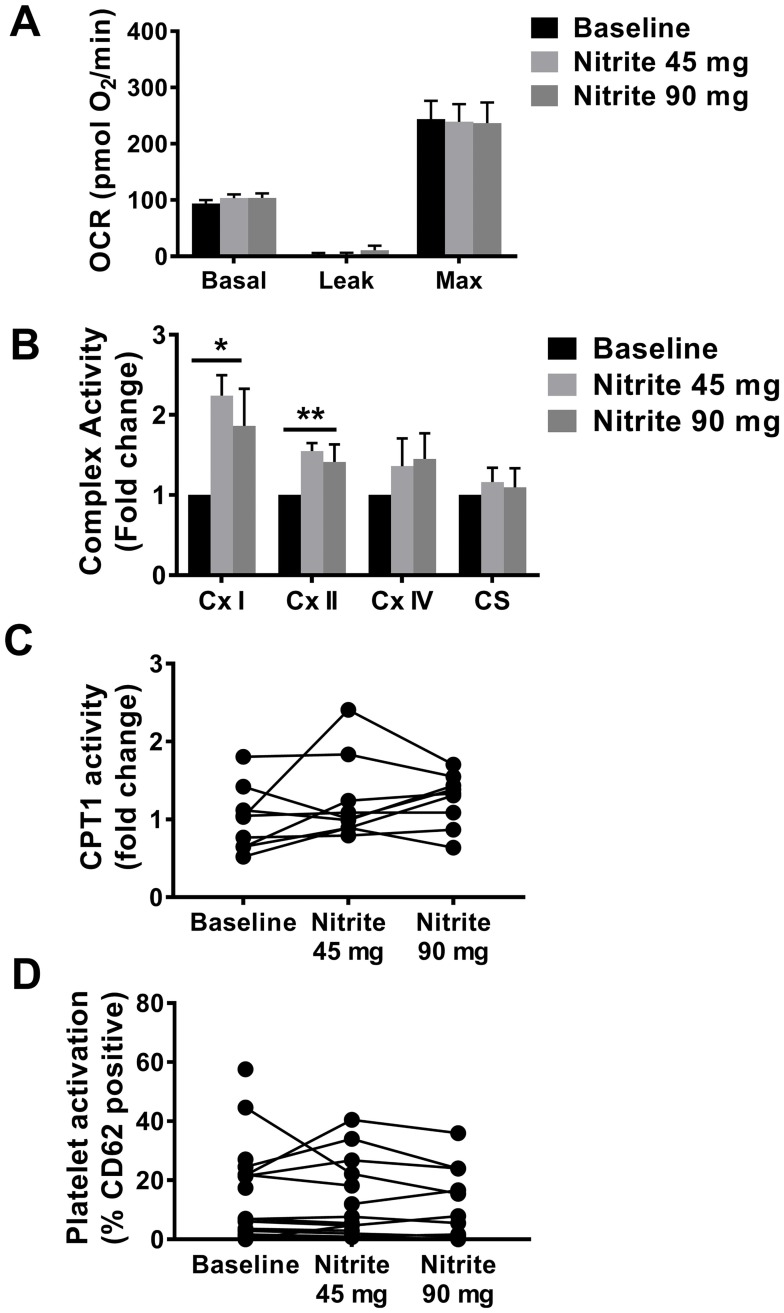
Nitrite does not affect Group 2 PH platelet respiration. **(A)** Quantification of individual components of the platelet OCR profile in Group 2 PH patients (n = 16) at baseline and after inhaled nitrite. Data are mean ± SEM. **(B)** Enzymatic activity (fold change of control) of mitochondrial complexes (Cx) I, II, IV, and citrate synthase (CS) from Group 2 PH platelets (n = 6–12) at baseline and after inhaled nitrite. Data are mean ± SEM. One-way ANOVA was used to compare groups. **(C)** Enzymatic activity of CPT1 in Group 2 PH platelets (n = 9) at baseline and after inhaled nitrite. **(D)** Platelet activation, as measured by CD62 positivity in Group 2 PH platelets (n = 17) at baseline and after inhaled nitrite. One-way ANOVA was used to compare groups. *#p* < 0.1, *p < 0.05, **p < 0.01.

Inhaled sodium nitrite was shown to improve hemodynamic parameters in Group 2 HFpEF-PH patients, specifically decrease in right atrial pressure (RAP), pulmonary capillary wedge pressure (PCWP), right ventricular (RV), and pulmonary artery (PA) pressures as well as pulmonary capillary compliance [22]. To determine whether a bioenergetic correlate accompanied these hemodynamic changes, platelet metabolism was measured in Group 2 PH patients at baseline and following 45 mg and 90 mg of inhaled sodium nitrite. Nitrite administration was not associated with a change any parameter of OCR in Group 2 PH patients (Fig 4A).

Comparison of ETC enzyme activities in Group 2 PH platelets at baseline and following 45 mg and 90 mg nitrite respectively revealed a 2.24 ± 0.26-fold and 1.86 ± 0.47-fold increase in complex I activity (*p* = 0.012, Fig 4B) and a 1.55 ± 0.1-fold and 1.41 ± 0.22-fold increase in complex II activity (*p* = 0.006, Fig 4B). There was a trend toward increased CPT1 enzyme activity after nitrite (1.24 ± 0.18-fold in 45 mg and 1.23 ± 0.11-fold in 90 mg compared to baseline, *p* = 0.17, Fig 4C). Platelet activation was not significantly altered in Group 2 PH platelets following nitrite inhalation (*p* = 0.84, Fig 4D).

## Discussion

The primary goal of this study was to determine whether platelets from subjects with Group 2 HFpEF-PH display bioenergetic changes compared to healthy subjects and whether these alterations are identical to those of Group 1 PH. We demonstrate that unlike Group 1 PH, platelets from Group 2 PH patients do not show a change in glycolytic rate compared to healthy controls. However, they did show a significant increase in maximal respiratory capacity similar to Group 1 subjects. Notably, this enhanced maximal respiration negatively correlated with patient RV hemodynamics. Further, despite hemodynamic improvement following inhaled nitrite administration in Group 2 PH patients, platelet respiration was unchanged by nitrite.

We previously demonstrated that platelets from patients with WHO Group 1 PAH demonstrated increased basal glycolytic rate and enhanced mitochondrial respiratory reserve, which was supported by increased FAO. Furthermore, others have shown enhanced glycolysis in HFpEF myocardium [27]. Here we observe that WHO Group 2 HFpEF-PH platelets showed enhanced platelet maximal OCR similar to Group 1, which was significantly attenuated by the inhibition of FAO, but no change in glycolytic rate compared to healthy controls. The lack of increased glycolysis in Group 2 platelets suggest a divergence between the metabolic dysfunction in Group 1 versus Group 2 PH and could be due to a number of etiological differences between the groups. For example, a major difference between these groups is the older age of Group 2 patients (Table 1). Aging has been associated with increased glycolytic rate in a number of tissues [28–31], thus it is possible that glycolysis is already significantly increased to a maximal level in the healthy age-matched controls, obscuring the difference between controls and Group 2 PH.

Interestingly, platelets from both Group 1 and Group 2 PH subjects showed an increase in maximal respiratory capacity that is attenuated by FAO inhibition. While Group 1 PAH platelets also showed an increase in the activity and expression of CPT1 [15], which facilitates fatty acid transport into the mitochondrion, this was not observed in Group 2 platelets. It is possible that Group 2 subjects, due to their potential higher degree of adiposity, may already have maximized fatty acid transport into the mitochondrion and increased FAO is mediated by increases in downstream beta oxidation enzymes. Future studies will investigate the FAO pathway in these subjects in more detail. Notably, in other cell types, increased respiratory capacity has been associated with resistance to oxidative stress and apoptosis [32]. This may have mechanistic implications in pulmonary vascular smooth muscle and endothelial cells for the pathogenesis of PH if these cell types show parallel changes in bioenergetics to platelets. While we did not explicitly investigate whether platelet bioenergetics parallel other vascular cell function in this study, prior work by our lab has shown that human platelets may serve as a bioenergetic surrogate for airway cells [33]. More study in both human and animal models is ongoing to clarify this point in Group 2 PH.

In a prior report, we showed that platelets from patients with Group 1 PAH had increased mitochondrial respiratory capacity which paralleled PAH hemodynamic severity, as indicated by higher mPAP, PVR, and RV SWI [15]. Altered platelet mitochondrial function can be hypothesized to develop as platelets are subjected to the hemodynamic derangements within the pulmonary circulation. Based on this hypothesis, we anticipated that Group 2 PH platelets would exhibit more pronounced mitochondrial alterations with increasingly severe hemodynamics. Like Group 1 PAH platelets, Group 2 PH platelets also displayed enhanced respiratory reserve compared to controls. However, we found no correlation between mPAP or PVR with Group 2 PH platelet reserve mitochondrial respiratory capacity, and no bioenergetic differences between isolated post-capillary PH platelets and combined post and pre-capillary PH platelets. Thus, we concluded that factors beyond pulmonary vascular hemodynamics likely influence platelet mitochondrial function in PH.

Because the different WHO classes of PH represent distinct disease states, platelet biology within each group may also be unique. While exact role of platelets in the pathogenesis of Group 1 PAH is not fully elucidated, aberrant signaling is known to occur in PAH between platelets and other cells within the pulmonary vasculature producing vasoconstriction, vascular remodeling, and thrombosis [34–36]. Hence, abnormal platelet bioenergetics in Group 1 PAH likely reflect intrinsic platelet dysfunction specific to this disease state which becomes more pronounced as PAH progresses. By contrast, WHO Group 2 patients are classically thought to develop elevated pulmonary pressures secondary to passive pulmonary venous congestion from left heart dysfunction. In addition to left heart disease, as exemplified by our cohort (Table 1), HFpEF patients have other significant underlying systemic disease, including obesity, hypertension, and diabetes, all of which are linked to platelet dysfunction [37–39] as well as systemic metabolic dysregulation [40, 41]. Thus, our finding of enhanced respiratory capacity in HFpEF-PH platelets likely reflects the convergence of multiple signals upon the platelet to produce a change in mitochondrial function.

RV function is an important predictor of outcomes in WHO Group 1 PAH [42, 43] as well as in left heart failure with reduced and preserved ejection fraction [44–46]. We previously found Group 1 PAH platelet metabolism to be associated with increased RV SWI, a measure of RV work and contractility which is decreased in PAH patients with poor outcomes [47] and improves in PAH patients treated with prostanoids [48]. In PAH, the increase in platelet metabolism paralleled other hemodynamic parameters of PAH severity and therefore was expected to track with increasing RV SWI as the RV compensates for higher workload. Because Group 2 PH pulmonary vascular hemodynamics did not correlate with platelet mitochondrial respiration, we did not anticipate an association between RV function and platelet metabolism. However, we were surprised to find a negative association between Group 2 PH RV SWI and platelet OCR wherein patients with lower RV work and contractility had greater aberration in platelet mitochondrial respiration. We therefore speculated that, in Group 2 PH, the apparent changes in platelet metabolism could reflect a disease state that is associated with RV dysfunction. Indeed, patients with HFpEF-PH have features of the metabolic syndrome such as insulin resistance and obesity (Table 2), which are associated with systemic inflammatory and neurohormonal dysregulation [49–51], which, in turn, promote intrinsic myocardial dysfunction [52]. Consequently, RV dysfunction in HFpEF-PH arises not just from increased pulmonary vascular load, but also from intrinsic cardiomyopathy in the setting of the metabolic syndrome. Aberrations in platelet activation and hemostasis are well described in the metabolic syndrome [37, 39], but to our knowledge, platelet bioenergetics have not been studied specifically in this context. We demonstrated that diabetic subjects tended to have higher platelet reserve OCR (Fig 3E), thus linking platelet metabolism to diabetes and insulin resistance, key features of the metabolic syndrome. We additionally found that obesity, another key feature of the metabolic syndrome, tended to correlate with increased platelet respiration (Fig 3D). Although not reaching statistical significance, likely due to underpower, these data suggest that the metabolic syndrome may contribute to altered platelet bioenergetics in this cohort.

Nitrite is a potent hypoxic vasodilator that mediates its effect through its reduction to nitric oxide (NO) and has also been shown to attenuate development of HFpEF in animal models through the activation of AMP kinase [53]. A major finding of this study was that nitrite produced no change in WHO Group 2 PH platelet bioenergetics despite alterations in hemodynamics in these patients. These data corroborate the fact that in Group 2 PH patients platelet respiration showed no association with hemodynamic measures such as mPAP, arguing further that the mitochondrial changes in Group 2 PH are likely not completely secondary to hemodynamic changes. However, acute nitrite or pure NO during hypoxia are known to inhibit mitochondrial respiration through its binding to complex IV (cytochrome c oxidase) [26, 54]. Thus, it is surprising that nitrite had no effect on platelet respiration. Others have shown that platelets from patients with heart disease including HFpEF, exhibit resistance to NO’s inhibitory effect on platelet hyperactivation and aggregation [55–58]. In patients with heart disease, platelet NO resistance is postulated to arise from NO scavenging by superoxide [59] or impaired sGC function [60, 61] and, interestingly, is associated with poor outcomes [62]. Platelet NO resistance may therefore provide an additional explanation for the unchanged platelet mitochondrial respiration in nitrite-treated HFpEF-PH patients despite an apparent hemodynamic effect within the blood vessels.

## Conclusions

Here we show that platelets from Group 2 PH patients demonstrate an alteration in bioenergetics compared to healthy controls. This alteration is characterized by an increase in respiratory capacity, similar to Group 1 PH subjects, but not by an increase in glycolysis. Further, nitrite, a potent modulator of hemodynamics, did not have any effect on platelet mitochondrial respiration. These data start to distinguish the metabolic phenotypes of Group 1 and Group 2 PH and may have important implications for delineating the hemodynamic versus non-hemodynamic modulation of mitochondrial function.
